# Editorial: Rising stars in comparative and clinical medicine: 2022

**DOI:** 10.3389/fvets.2023.1297462

**Published:** 2023-10-20

**Authors:** Jiadong Chen, Yaru Ji, Khalid Mehmood, Md. Masudur Rahman, Isa Ozaydin, Hui Zhang, Kun Li

**Affiliations:** ^1^MOE Joint International Research Laboratory of Animal Health and Food Safety, College of Veterinary Medicine, Nanjing Agricultural University, Nanjing, China; ^2^Faculty of Veterinary and Animal Sciences, The Islamia University of Bahawalpur, Bahawalpur, Pakistan; ^3^Faculty of Veterinary, Animal and Biomedical Sciences, Sylhet Agricultural University, Sylhet, Bangladesh; ^4^Faculty of Veterinary Medicine, Kafkas University, Kars, Türkiye; ^5^College of Veterinary Medicine, South China Agricultural University, Guangzhou, Guangdong, China

**Keywords:** livestock, poultry, microbiota, drug resistance, probiotics, toxicity

## Introduction

The main aim of this Research Topic is to establish a platform for the sharing of research findings in the field of comparative and clinical medicine. Many research queries and recommendations have been offered as components of this Research Topic by promising talents in the field of clinical medicine. In this special e-collection, there are 28 papers covering the various aspects of clinical and comparative medicine. Most of the studies featured in this compilation can be classified into the following research domains: (1) Animal diseases and their management; (2) Drug resistance and alternative strategy; (3) Microbiota in the context of health and diseases; and (4) Poultry diseases and toxicological studies.

## Animal diseases and their management

Abdullah et al. carried out a study on molecular detection of *Theileria* spp., a significant prevalence of *Theileria* infection was revealed in native sheep, affirming the endemic nature of the disease in the investigated region. In traditional husbandry practices, animals that were sub-clinically infected might continue to serve as a source for infecting ticks. The impact of theileriosis on metabolic disruptions of different systems and organs of the host body is evidenced by alterations in specific biochemical and hematological profiles in affected sheep (1). The disease is economically important because of its association with the elevated level of morbidity and mortality in sheep and attributing to significant economic losses in the livestock sector (2).

Naeem et al. studied the molecular prevalence of *Anaplasma ovis* in sheep. Blood samples tested from sheep in Punjab province of Pakistan revealed a moderate prevalence of *A. ovis*. Specific susceptibility to *A. ovis* was not observed in any of the enrolled sheep breeds in the study. Data generated in this study might support the development of prophylactic detection methods and integrated control strategies against tick-borne diseases in sheep.

Qamar and Alsayeqh conducted a review on foodborne *Toxoplasma gondii* infections. Developed nations exhibit a lower prevalence of food-related disorders compared to third-world countries (3). According to their review, it was observed that animals and pets served as both parasite reservoirs and source of *T. gondii* infections in humans. Most of the diagnostic techniques employed in the studies under observation primarily included serological tests, with only a limited number of studies utilizing molecular tools. To gain a precise understanding of the disease pattern and to control the disease efficiently, it is advisable to adopt advanced molecular diagnostic tools, given their greater reliability.

Alvi and Alsayeqh performed a review on echinococcosis, a zoonotic disease transmitted through food, with a particular emphasis on its epidemiology. Echinococcosis is a neglected parasitic disease listed by the WHO, and it is caused by various species within the *Echinococcus* genus (4). Worldwide prevalence characterizes this disease, with significant economic losses incurred among the farmers and the development of cystic disease in unintentional human hosts (5). This review article provides a concise overview of taxonomy, a brief historical account, the extent of economic losses, the range of hosts and life cycle, factors contributing to risk, and clinical manifestations of echinococcosis. Additionally, the review compiles the prevalence of echinococcosis across various continents using copro- and sero-ELISA-based methods. Existence of genetic variations within species and their correlation with the virulence of the parasite demands the identification of specific markers to be used for clinical applications toward the diagnosis and control of the disease in the future.

Rashid et al. evaluated the cost-effectiveness of anthelmintic treatment and its comparative efficacy in commercial dairy farms. Intestinal parasitic infections posed significant challenges in achieving optimal production and ensuring the health and welfare of animals, including cattle and buffaloes. Utilizing anti-parasitic treatments seemed to be a dependable approach (6). Nevertheless, the efficacy and choice of suitable anthelmintics must be assessed based on local conditions. Overall, this enhanced production efficiency results in a favorable and direct cost-benefit ratio for dairy farmers.

Avberšek et al. studied the identification of pathogenic skin bacteria in dogs and cats through visual means. The diagnostic cytology was complemented by the use of chromogenic culture media. Skin infections caused by bacteria, such as otitis externa and pyoderma, were frequently observed in dogs and cats brought to the veterinary clinic. The chromogenic media incorporation in animal hospitals could substantially enhance the diagnostic capabilities for identifying skin problem by the species of pathogens in ~80% of cases.

Teoh et al. reported that single-nucleotide polymorphisms (SNPs) associated with inflammatory bowel disease (IBD) leads to an elevation in local thyroglobulin expression, contributing to the development of inflammation in miniature dachshunds (MDs). In MDs, Inflammatory Colorectal Polyp (ICRP) represents a chronic form of IBD. It is distinguished by granulomatous inflammation involving neutrophil infiltration and hyperplasia of goblet cells in the colon. As a result, TG emerges as a potential diagnostic target for ICRP in MDs, and the targeting of TG-mediated activation of the IL-6 amplifier in the colon is potentially a viable therapeutic approach for ICRP.

Pila et al. studied assessing of B-cell lymphoma 2 (Bcl-2) as a predictor for chronic kidney disease (CKD) in cats where they found lowered Bcl-2 levels in CKD cats compared to age-matched healthy cats. These lower levels were linked to higher BUN, creatinine, and greater CKD severity. The study suggested that Bcl-2 might be helpful in differentiating CKD-afflicted cats from healthy ones.

Ahmed et al. studied the effects of adding β-mercaptoethanol (βME) and epidermal growth factor (EGF) to buffalo embryos produced *in vitro*. Their findings showed that adding EGF improved buffalo embryo growth and development, while βME had a lesser effect. βME stimulated embryo growth when added during maturation and fertilization. However, the combination of EGF (20 ng/mL) and βME (50 μM) did not significantly enhance buffalo embryos compared to using each one separately.

Zeeshan et al. performed a sero-epidemiological study on abortion-causing bacterial agents in small ruminants. According to the study, fetal losses in small ruminants were primarily caused by *Brucella* spp., *Coxiella burnetiid*, and *Chlamydia abortus* (7). In the study area, the prevalence of brucellosis and coxiellosis was relatively higher compared to chlamydiosis. Binary logistic regression revealed a substantial link between ticks and brucellosis and coxiellosis.

Parvin et al. investigated respiratory diseases epidemics of poultry in Bangladesh using two real-time PCR-based simultaneous detection assays. Respiratory microorganisms posed a significant risk for poultry sector in Bangladesh, with Infectious Bronchitis Virus (IBV), Newcastle Disease Virus (NDV) and Avian Influenza viruses (LPAI H9N2 and HPAI H5N1) being common culprits among chicken flocks. The utilization of TaqMan and SYBR Green multitarget simultaneous RT-qPCR assays in screening outbreak samples (tissues and swabs) proved effective, offering accurate, swift, and reproducible results.

## Drug resistance and alternative strategy

Alvi et al. uncovered new population diversity and benzimidazole resistance in *Echinococcus granulosus* from bovine sources using novel *cytb* and *nad5* genes. Cystic echinococcosis (CE) is an overlooked zoonotic illness induced by *Echinococcus granulosus* (8). The parasite impacts a broad spectrum of domesticated animals and wildlife (4). The research revealed that the *Echinococcus* species exhibited genetic diversity using NADH dehydrogenase subunit 5 (nad5) and mitochondrial cytochrome b (cytb) genes. This investigation provides insights into the prevalence of benzimidazole resistance in Pakistani *Echinococcus granulosus*.

Lee et al. studied analysis of fluoroquinolone usage and the identification of high-level ciprofloxacin resistance in *Enterococcus faecalis* isolated from integrated broiler operations in South Korea. The study confirmed the widespread presence of HLCR *E. faecalis* in Korean broiler operations. As a result, it is essential to reduce the prevalence of resistant bacteria through regulatory measures such as farm environment cleaning and disinfection. Additionally, there is a need to minimize antimicrobial usage by prescribing them according to scientific proof of disease instead of solely action for disease prevention.

Aqib and Alsayeqh reviewed vancomycin resistance, a growing concern for both public health and animal (9). These resistant genes, collectively referred to as the “Van cluster”, were harbored by a variety of microbes, which in turn exchanged them among themselves. Among animals, humans, and the environment, resistance transfer was widely observed, with the potential for both reverse zoonosis and zoonosis. Relevant cost-effective losses, as well as risks to public health, farm animal health, food security and food safety were posed by this situation.

Ul Haq et al. studied on the enhancement of β-lactam and fluoroquinolones antibiotics' effectiveness using artemisinin and its derivatives against multi-drug resistant (MDR) *Escherichia coli*. Time-kill assays revealed that these synergistic combinations exhibited significant effectiveness in the early hours of incubation, indicating their potential utility during outbreaks. The study suggests that, alongside the development of new antibiotics, improvements can be made to existing ones by incorporating enhancers such as artemisinin and its derivatives.

Altaf and Alkheraije showed that cell membrane-coated nanoparticles might be considered as an emerging antibacterial approach for pathogens in food animals. Traditional methods for fighting against infections primarily relied on antibiotics. However, the rise of drug-resistant bacteria and the increasing prevalence of deadly bacterial infections had underscored the need for new therapeutic agents against the infectious diseases. Recent progress in nanotechnology has led to the development of various nanoscale nanoparticles enclosed within cell membranes. Significant efforts have focused on enhancing nanoparticle functionality and protecting them during interactions with pathogens or exo/endotoxins.

Manan et al. made an investigation aiming to alter drug resistance in emerging milk-borne pathogens using antibiotics and nanoparticles based on sodium alginate. The study observed a rise in the drug resistance and occurrence of *S. agalactiae* and *K. pneumoniae*. It was noteworthy that nanoparticles and antibiotics stabilized with sodium alginate exhibited strong antibacterial properties against both the bacterial strains. Specifically, the formulation containing nanoparticles of MgO and antibiotics within a gel (sodium alginate) displayed an enhanced antibacterial effect. Notably, the cytotoxicity of the nanoparticles of MgO used in this investigation was considerably less than the positive control.

Abdel Ghfar et al. studied the impact of *Nigella sativa* and *Allium sativum* on the mitigation of aluminum toxicity in albino rats. It was concluded that aluminum had detrimental effects on the health of both animals and humans. Treatment with *Allium sativum* and *Nigella sativa* was found to alleviate the adverse effects of aluminum and facilitate the restoration of the liver, kidney, and testis to their normal state. Furthermore, the effectiveness of *Nigella sativa* and *Allium sativum* in combating aluminum toxicity was confirmed by the blood biochemical results in this study.

Zia and Alkheraije studied on recent developments in utilizing bacteriophages as substitutes for antimicrobials against pathogens in food animals. The foremost challenges in the global food industry continued to revolve around food safety and sustainability (10). Bacteriophages, natural antibacterial agents, had effectively worked on various established and emerging foodborne pathogens (11). While bacteriophages (phages) are widespread in the environment and pose no harm to humans or animals, they are well-suited for detecting and managing pathogens throughout the food supply chain. Their inherent capacity to infect and eliminate specific bacteria makes them a potent tool for both detection and control purposes. Instead of relying on chemical preservatives, biological approaches like bacteriophage biocontrol can be employed to address food contamination, even though their antimicrobial effectiveness in food systems may be diminished compared to lab conditions (12).

Guo et al. performed an *in-vitro* assessment of the immunomodulatory effects of sulphation-modified total ginsenosides derivative-3 (SMTG-d3). According to the study, TG and SMTG-d3 were found to enhance the activity of peritoneal macrophages, NK cells and T lymphocytes, along with promoting the production of TNF-α and IFN-γ during the anti-tumor process. Therefore, utilizing the active components of ginseng as a foundation and exploring the relationship between structural changes and activity (toxicity) could be used to develop novel therapeutic target molecules.

## Microbiota in the context of health and diseases

Zhu et al. performed a comparative study on gut fungus structure and makeup in yaks fed on different feeding models. Specifically, the researchers compared and assessed the diversity of the intestinal fungus species in grazing domestic yaks (GYG), house-fed domestic yaks (HFG) and wild yaks (WYG). The findings showed that Ascomycota and Basidiomycota were the dominant phyla in the gut fungus spices, regardless of the feeding models. Fungal taxonomic analysis identified significant differences in 20 genera among GYG and WYG, as well as 16 genera between WYG and HFG. In summary, this research demonstrated that the structure and makeup of gut fungi varied significantly among yaks raised in different breeding groups.

Li et al. performed a study on *Bacillus subtilis* from yaks and its probiotic qualities through complete genome analysis. Probiotics offer various health benefits to hosts and are of interest. Yaks in the Tibetan plateau are known for their disease resistance and resilience, possibly due to their internal probiotics. The research pinpointed genes related to anti-oxidation and biological adhesion in the *Bacillus subtilis* genome. This study highlighted *Bacillus subtilis*' beneficial properties from a genomic angle, raising awareness of its potential and laying the groundwork for probiotic product development.

Lan et al. performed a study on the shifts in the intestinal fungus community in horses at different health conditions. The results revealed significant alterations in both the diversity and composition of the gut fungus spices in horses with diarrhea. There were notable changes in the types and in diarrheal horses. Furthermore, the analysis identified 175 distinct fungal genera that differed between the intestinal fungus communities of infected and healthy horses, with four fungal genera increasing significantly and 171 bacterial genera decreasing significantly during diarrhea. Previous researches also highlighted the value of comparing equine gut microbiota to gain insights into its vital role in animal health (13).

## Poultry diseases and toxicological studies

Saeed and Alkheraije reported that botanicals offered a promising method for managing cecal coccidiosis in poultry. Prior research had indicated that plants contained various botanical compounds, which varied in their quantities and proportions (14). These compounds exhibited anticoccidial and antioxidant characteristics against avian cecal coccidiosis (15, 16). Anticoccidial effects have been demonstrated by sulfur compounds, essential oils (terpenes and derivatives), saponins, phenolics and other compounds through various mechanisms. However, research on their potential toxicity concerning coccidiosis is lacking. Evaluating their toxicological profiles is essential before considering their therapeutic use to understand their interactions within the body.

Wang et al. observed notable changes in liver metabolism in chickens exposed to thiram, resulting in significant alterations in specific metabolites and metabolic pathways. These findings enhance our understanding of how thiram impacted broiler liver metabolism and emphasized the potential role of hepatic metabolic disorder. In conclusion, this study highlights the significant influence of thiram exposure on chicken liver metabolism and provides a foundation for regulating thiram use and disposal to protect environmental quality and poultry health.

Naz et al. studied on the induction of histopathological, serum biochemical, oxidative stress and clinico-hematological changes in freshwater fish rohu (*Labeorohita*) by copper sulfate. Significant modification in serum biochemical and hematological parameters, along with alterations in histopathological conditions in *Labeorohita*, were observed in this study. As a result of these findings, further research is suggested to determine the optimal CuSO4 dose for regular use in the decontamination of fish ponds.

Mashkoor et al. showed that arsenic and chromium exposure in broiler chicks resulted in oxidative stress and toxicity, leading to increased gross and microscopic lesion severity compared to other treatment groups. These contaminants negatively impacted on hematological and biochemical parameters. However, the administration of vitamin E and bentonite can mitigate the toxicity and oxidative stress induced by arsenic and chromium.

## Conclusion

Taken together, the articles published in our Research Topic make important contributions to understand that how livestock production can be improved with the application of various diagnostic and treatment options in clinical and comparative veterinary medicine. However, further research on these topics is required to develop an improved understanding of the use of alternative strategies for the treatment of various pathological disorders. The authors would like to thank all the contributors who participated in this Research Topic.

## Author contributions

JC: Conceptualization, Formal analysis, Methodology, Visualization, Writing—original draft. YJ: Conceptualization, Investigation, Software, Writing—review and editing. KM: Conceptualization, Funding acquisition, Resources, Visualization, Writing—original draft, Writing—review and editing. MR: Formal analysis, Project administration, Validation, Writing—review and editing. IO: Data curation, Methodology, Supervision, Writing—review and editing. HZ: Writing—review and editing, Funding acquisition, Investigation, Resources, Supervision. KL: Conceptualization, Project administration, Software, Validation, Writing—review and editing.
